# Apatinib as a third- or further- line treatment in patients with advanced NSCLC harboring wild-type EGFR

**DOI:** 10.18632/oncotarget.23612

**Published:** 2017-12-22

**Authors:** Shencun Fang, Meiling Zhang, Guihong Wei, Kai-Hua Lu

**Affiliations:** ^1^ Department of Respiratory Medicine Center, Nanjing Chest Hospital, Nanjing, Jiangsu, China; ^2^ Department of Oncology, The First Affiliated Hospital of Nanjing Medical University, Nanjing, Jiangsu, China; ^3^ Department of Respiratory and Critical Care Medicine, The First Affiliated Hospital of Nanjing Medical University, Nanjing, Jiangsu, China

**Keywords:** non-small-cell lung cancer, angiogenesis, apatinib, VEGFR-2

## Abstract

**Objectives:**

This study was conducted to evaluate the efficacy and safety of apatinib in advanced NSCLC patients with EGFR wild-type who have failed more than second-line chemotherapy.

**Materials and Methods:**

We retrospectively analyzed patients with EGFR wild-type advanced NSCLC who were treated with apatinib from January 2014 to August 2016. Objective response rate (ORR), disease control rate (DCR), progression free survival (PFS), overall survival (OS), and adverse events (AEs) were reveiwed and evaluated. Univariate and multivariate analyses were performed to determine the prognostic factors.

**Results:**

36 patients were evaluable for safety and efficacy. 6 patients obtained partial response, and 21 showed stable disease. The ORR and DCR were 16.7% and 75%, respectively. The median PFS and OS were 4.5 months and 8.2 months, respectively. Prognostic variable for a longer OS was good performance status (*p* = 0.015). Most adverse reactions were mild or moderate.

**Conclusions:**

Apatinib should be recommended as a third- or further- line therapy in advanced NSCLC patients with EGFR wild-type due to its better efficacy and tolerable toxicity.

## INTRODUCTION

Non-small-cell lung cancer (NSCLC) is the leading cause of cancer-related death worldwide [1]. With development of targeted therapy, epidermal growth factor receptor (EGFR) mutations were found to be one of the most common and important oncogenic drivers in patients with NSCLC. The tyrosine kinase inhibitors (TKIs), such as gefitinib, afatinib and erlotinib, are recommended as first-line treatment for advanced NSCLC patients harboring EGFR mutations [2]. However, for the majority of advanced NSCLC patients without identifiable driver oncogenes, platinum-based doublet chemotherapy is recommended as standard first-line treatment option [3]. Pemetrexed, docetaxel and erlotinib are currently recommended as standard second-line chemotherapy for advanced NSCLC based on clinical trials [4–6]. Though a number of patients with a favorable performance status require further salvage therapies, there is no definitive therapeutic regimen for third-line or beyond therapy for these patients.

Apatinib, a small molecule tyrosine kinase inhibitor of vascular endothelial growth factor receptor-2 (VEGFR-2), is a first-generation oral anti-angiogenesis drug approved by the China Food and Drug Administration (CFDA) for the treatment of advanced gastric cancer. Apatinib have also demonstrated encouraging antitumor activity in hepatocellular carcinoma [7], sarcomas [8] and breast cancer [9] in preclinical and clinical experiments. Preliminary results of an apatinib clinical trial presented at the ASCO meeting in 2012 showed potential survival benefits in advanced non-squamous NSCLC patients [10]. We have also reported that three advanced NSCLC patients with EGFR wild-type who received apatinib as post second-line therapy achieved partial response [11]. But to our knowledge, there is almost no detailed clinical data regarding the efficacy and safety of apatinib in advanced NSCLC. Herein, we retrospectively analyzed the outcome and toxicity of apatinib in advanced NSCLC patients with EGFR wild-type who have failed more than second-line chemotherapy.

## RESULTS

### Characteristics of patients

The demographic characteristics of 36 patients with advanced NSCLC were summarized in Table 1. The median age of the patients was 65 years, and there were more males (77.8%) than females (22.2%). Most patients had a favorable Eastern Cooperative Oncology Group (ECOG) performance status (0–1). 26 patients received apatinib as third-line therapy and 10 as further-line treatment. 27 patients had non-squamous cell carcinoma (adenocarcinoma: *N* = 25, poorly differentiated carcinoma: *N* = 2) and 9 had squamous cell carcinoma.

**Table 1:** Baseline characteristics of 36 patients treated with apatinib

Characteristic	Number (%)
Age (years)	
< 65	16 (44.4)
≥ 65	20 (55.6)
Gender	
Female	8 (22.2)
Male	28 (77.8)
Smoking history	
Smoker	15 (41.7)
Non-smoker	21 (58.3)
ECOG performance status	
0–1	25 (69.4)
2	11 (30.6)
pathological type	
Adenocarcinoma	25 (69.4)
Squamous carcinoma	9 (25)
Poorly differentiated	2 (5.6)
Location	
Central	16 (44.4)
Peripheral	20 (55.6)
Line of apatinib	
Third line	26 (72.2)
Further line	10 (27.8)

### Clinical efficacy

**Figure 1:**
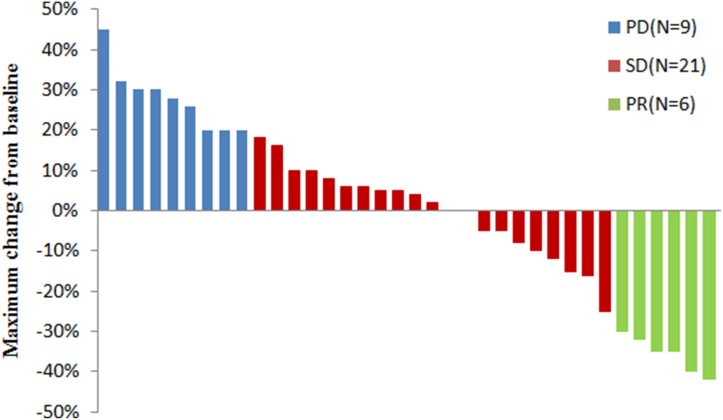
Maximum change in tumor size(target lesions) from baseline in patients with advanced NSCLC (*N* = 36)

**Figure 2:**
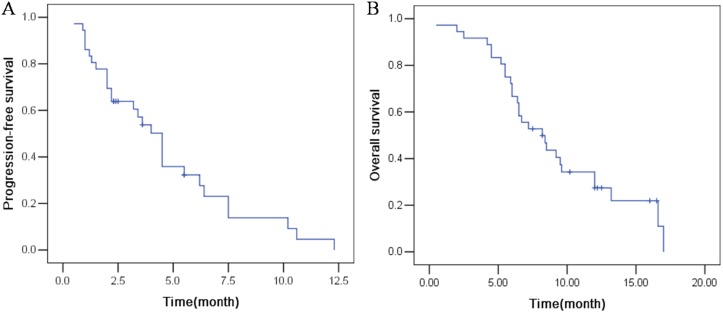
The efficacy evaluation of apatinib in patients with advanced NSCLC (**A**) Progression-free survival. (**B**) Overall survival.

As shown in the waterfall plot (Figure 1), none achieved a complete response, 6 patients obtained partial response, and 21 showed stable disease. The ORR and DCR were 16.7% and 75%, respectively. The median follow-up duration was 11.6 months, the median PFS was 4.5 months (95% CI, 2.2 to 6.2 months), and the median OS was 8.2 months (95% CI: 5.7–10.6 months) (Figure 2).

### Univariate and multivariate analyses

In univariate analysis, patients with good performance status (*p* = 0.026), malignant pleural effusion (*p* = 0.013), and peripheral NSCLC (*p* = 0.037) were associated with a longer PFS (Table 2). Good performance status (*p* = 0.012) and malignant pleural effusion (*p* = 0.016) were significant predictive factors for a longer OS (Table 3). However, in multivariate analysis, patients with good performance status (*p* = 0.023, HR:4.28, CI:1.22–10.01) and malignant pleural effusion (*p* = 0.01,HR:0.27, CI:0.1–0.73) had significantly longer PFS. Prognostic variable for a longer OS was only good performance status (*p* = 0.015, HR:3.98, CI:1.31–6.09).

**Table 2:** Progression-free survival in univariate and multivariate analysis

Variable	Median (95% CI)	*P* value	
		univariate	multivariate
Age (years)		0.232	
< 65	2.2 (1.81–2.59)		
≥ 65	6.4 (3.02–9.78)		
Gender		0.254	
Female	3.4 (0.87–5.93)		
Male	10.2 (0.6–19.8)		
Smoking history		0.234	
Smoker	3.4 (1.26–5.54)		
Non-smoker	10.2 (0–21.04)		
ECOG performance status		0.026	0.023 (HR:4.28, CI:1.22–10.01)
0–1	10.2 (2.13–18.23)		
2	3.4 (1.53–5.27)		
pathological type		0.909	
Adenocarcinoma	6.4 (0.07–12.73)		
Non-adenocarcinoma	3.4 (2.33–4.48)		
Line of apatinib		0.083	
3	10.2 (2.28–18.12)		
≥ 4	2 (0.92–3.09)		
malignant pleural effusion		0.013	0.01 (HR:0.27, CI:0.1–0.73)
Yes	6.4 (0.64–12.17)		
No	2 (1.42–2.58)		
Location		0.037	0.138 (HR:0.478, CI:0.1–1.37)
Central	2.2 (1.87–2.54)		
Peripheral	10.2 (4.53–15.87)		

**Table 3:** Overall survival in univariate and multivariate analysis

Variable	Median (95% CI)	*P* value	
		univariate	multivariate
Age (years)		0.232	
< 65	2.2 (1.81–2.59)		
≥ 65	6.4 (3.02–9.78)		
Gender		0.904	
Female	8.4 (6.07–10.73)		
Male	6.4 (5.59–7.23)		
Smoking history		0.962	
Smoker	6.5 (6.32–6.68)		
Non-smoker	8.4 (6.51–10.29)		
ECOG performance status	0.012		0.015 (HR:3.98, CI:1.31–6.09)
0–1	12 (6.03–17.97)		
2	6.5 (5.73–10.67)		
pathological type		0.312	
Adenocarcinoma	9.5 (7.75–11.25)		
Non-adenocarcinoma	6.5 (5.98–7.02)		
Line of apatinib		0.595	
3	8.2 (4.89–11.51)		
≥ 4	7.2 (3.96–10.44)		
malignant pleural effusion		0.016	0.087 (HR:0.47, CI:0.2–1.12)
Yes	12 (7.76–16.24)		
No	6.4 (5.71–7.09)		
Location		0.589	
Central	6.5 (2.77–10.23)		
Peripheral	9.2 (5.9–12.5)		

### Toxicity

Most adverse reactions were mild and controllable (Table 4). A total of four patients were treated with a reduced apatinib dose of 250 mg/day resulting from hypertension and hand-foot syndrome. The most common AEs of all levels were hypertension (55.6%), hand-foot syndrome (30.5%) and Proteinuria (22.2%). The most frequently observed AEs of grade 3 were as follows: hypertension (16.7%), hand-foot syndrome (11.1%), proteinuria (5.6%), neutropenia (2.8%), and thrombocytopenia (2.8%). No grade 4 AEs or treatment-related deaths were observed in this study.

**Table 4:** Adverse events in the apatinib treatment

Adverse event	Grade 1-2 (*n*, %)	Grade 3 (*n*, %)	Total (*n*, %)
Neutropenia	4 (11.1)	1 (2.8)	5 (13.9)
Anemia	3 (8.3)		3 (8.3)
Thrombocytopenia	2 (5.6)	1 (2.8)	3 (8.3)
Hypertension	14 (38.9)	6 (16.7)	20 (55.6)
Hand-foot syndrome	7 (19.4)	4 (11.1)	11 (30.5)
Proteinuria	6 (16.7)	2 (5.6)	8 (22.2)
Mucositis	4 (11.1)		4 (11.1)
Nausea	3 (8.3)		3 (8.3)
Fatigue	2 (5.6)		2 (5.6)
Elevated transaminase	3 (8.3)		3 (8.3)
Hyperbilirubinemia	1 (2.8)		1 (2.8)
Hemoptysis	1 (2.8)		1 (2.8)
Anorexia	3 (8.3)		3 (8.3)
Testicular swelling	1 (2.8)		1 (2.8)
Diarrhea	1 (2.8)		1 (2.8)

## DISCUSSION

Angiogenesis is one of the hallmarks of cancer and plays a critical role in the growth, progression, and metastasis of solid malignancies, including NSCLC. Activation of angiogenesis depends on the balance between pro- and anti-angiogenesis factors. Among these factors, vascular endothelial growth factor (VEGF) family is the principal mediator involved in the angiogenic pathway [12]. The binding of VEGF to VEGFR-2, a key factor in the cancer angiogenic process, induces activation of the downstream molecules of VEGFR-2 and results in subsequent effects on the vascular endothelium, including increased cellular permeability, proliferation, and migration necessary for angiogenesis [13]. Therefore, blockage of VEGFR-2 could be a promising treatment for a variety of malignancies.

Apatinib, a novel small-molecule oral VEGFR-2 inhibitor, have been demonstrated to have encouraging antitumor activity in a variety of tumors. A multicenter Phase II study demonstrated that apatinib signifcantly prolonged OS and PFS in patients with advanced breast carcinoma patients who who failed third-line or beyond treatment [14]. Apatinib also exhibited objective efficacy in stage IV sarcoma patients who failed in chemotherapy [8]. A retrospective study was conducted to evaluate the efficacy of apatinib as salvage treatment in advanced NSCLC. The results showed that the DCR and ORR were 61.9% and 9.5%, respectively [15].

In this retrospective study, we report the first study of apatinib as third- or further- line treatment in advanced NSCLC patients harboring wild-type EGFR to evaluate its efficacy and safety. The results demonstrated the efficacy of apatinib as shown by the ORR of 16.7% and DCR of 75% in 36 patients, which was superior to that of single-agent chemotherapy in the third-line setting. For example, in two retrospective studies, the ORR and DCR from third- or further- line pemetrexed treatments were 7.4–16.3% and 42.1–53.6%, respectively [16, 17]. Moreover, Harada et al. [18] published the results from a Phase II trial in which patients who failed second-line treatment received amrubicin as a rescue therapy. Of the 41 enrolled patients, four patients (10%) had a PR, and 21 patients (51%) showed SD for an overall DCR of 61%. Finally, vinorelbine as a third-line therapy had limited activity in advanced NSCLC, with the ORR of 11% and DCR of 31% [19].

The survival data in our study demonstrated that the median PFS and OS were 4.5 months and 8 months, respectively, which was similar to other reports in third-line chemotherapy [17, 19, 20]. However, the median PFS and OS were longer in our study than those reported by Song et al. [15], who demonstrated that the median PFS and OS for apatinib treatment in advanced NSCLC were 4.2 and 6.0 months, respectively. A reasonable explanation for this beneficial outcome may be attributed to a higher proportion of patients with good performance status in our study. Another contributing factor might be that patients with EGFR mutations were excluded from our study.

Apatinib 750 mg/day was recommended for the treatment of metastatic breast cancer in a Phase IIa trial, but dose modifications caused by serious adverse events were very common [14]. Therefore, 500 mg/day was subsequently prescribed in the phase IIb study. In the present study, apatinib 500 mg/day was also started as the initial dose, and the results demonstrated that most adverse reactions were mild or moderate. The most frequently side effects were hypertension, hand–foot syndrome, and proteinuria, which were consistent with those reported in other studies [8, 14, 15, 21] .

In summary, apatinib should be recommended as a third- or further- line therapy in advanced NSCLC patients with EGFR wild-type due to its better efficacy and tolerable toxicity, especially in patients with good performance status. However, further prospective studies are warranted to define the efficacy and safety of this treatment.

## MATERIALS AND METHODS

### Patients

From 1 January 2014 to 30 August 2016 in Nanjing Chest Hospital, 36 advanced NSCLC patients with EGFR wild-type who failed more than second-line chemotherapy received apatinib as third-line or further treatment. All patients had been cytologically or histologically diagnosed with advanced NSCLC. Detailed variables of age, gender, smoking history, pathological type, metastasis sites, objective response rate and other clinical data were obtained from electronic medical record system. The initial dose of apatinib was 500 mg/day and and the dose should be reduced to 250 mg if there is an untolerated toxicity. Written informed consent was obtained from all patients and this study was approved by the medical ethics committee of the Nanjing Chest Hospital (2016-KL001-03).

### Efficacy and safety assessments

Tumor response was evaluated by computed tomography scans according to the Response Evaluation Criteria in Solid Tumor Criteria Version 1.1. Progression-free survival (PFS) was defined as the time from the first administration of apatinib to the date of disease progression. Overall survival (OS) was defined as the time from the first administration of apatinib to death or loss of follow-up. Complete response (CR) means disappearance of all target lesions. Partial response (PR) means the longest diameter of target lesion was reduced by at least 30%. Progressive disease (PD) means that the longest diameter of the target lesion increases by at least 20%, or the appearance of new lesion. Stable disease (SD) means the longest diameter of the target lesion increased to less than PD, or reduced to less than PR. disease control rate (DCR) = (CR+PR+SD ) / total number of cases × 100%, and the objective response rate (ORR) = (CR+PR) / total number of cases × 100%. Adverse events (AEs) were determined by the National Cancer Institute Common Toxicity Criteria for adverse events version 4.0.

### Statistical analyses

OS and PFS were assessed using the Kaplan–Meier method and compared using the log-rank test. Multivariate analysis of the independent prognostic factors was evaluated using the Cox regression model. Statistical analyses were performed using SPSS version 19.0. *P* < 0.05 were considered to be statistically significant.
